# Dimerisation of HIV-2 genomic RNA is linked to efficient RNA packaging, normal particle maturation and viral infectivity

**DOI:** 10.1186/1742-4690-4-90

**Published:** 2007-12-13

**Authors:** Anne L'Hernault, Jane S Greatorex, R Anthony Crowther, Andrew ML Lever

**Affiliations:** 1 Department of Medicine, University of Cambridge, Addenbrooke's Hospital, Cambridge CB2 2QQ, UK; 2 MRC Laboratory of Molecular Biology, Cambridge CB2 0QH, UK

## Abstract

**Background:**

Retroviruses selectively encapsidate two copies of their genomic RNA, the Gag protein binding a specific RNA motif in the 5' UTR of the genome. In human immunodeficiency virus type 2 (HIV-2), the principal packaging signal (Psi) is upstream of the major splice donor and hence is present on all the viral RNA species. Cotranslational capture of the full length genome ensures specificity. HIV-2 RNA dimerisation is thought to occur at the dimer initiation site (DIS) located in stem-loop 1 (SL-1), downstream of the main packaging determinant. However, the HIV-2 packaging signal also contains a palindromic sequence (pal) involved in dimerisation. In this study, we analysed the role of the HIV-2 packaging signal in genomic RNA dimerisation *in vivo *and its implication in viral replication.

**Results:**

Using a series of deletion and substitution mutants in SL-1 and the Psi region, we show that in fully infectious HIV-2, genomic RNA dimerisation is mediated by the palindrome pal. Mutation of the DIS had no effect on dimerisation or viral infectivity, while mutations in the packaging signal severely reduce both processes as well as RNA encapsidation. Electron micrographs of the Psi-deleted virions revealed a significant reduction in the proportion of mature particles and an increase in that of particles containing multiple cores.

**Conclusion:**

In addition to its role in RNA encapsidation, the HIV-2 packaging signal contains a palindromic sequence that is critical for genomic RNA dimerisation. Encapsidation of a dimeric genome seems required for the production of infectious mature particles, and provides a promising therapeutic target.

## Background

Retroviruses encapsidate two copies of the positive sense single-stranded genomic RNA. Encapsidation is very specific, as the virus has to select and package the full length genomic RNA over the vast excess of cellular and subgenomic RNA species. In human immunodeficiency virus type 1 (HIV-1), this mechanism is well understood. An RNA motif, downstream of the viral splice donor and upstream of the Gag start codon, interacts with the Gag structural protein and ensures specificity of encapsidation for full length rather than spliced RNAs [1-6].

In the case of HIV-2, the process is less well understood as the main packaging determinant (Psi or Ψ) appears to be located upstream of the major splice donor [7]. In a study to further map the HIV-2 encapsidation signal, a 28 nucleotides (nt) sequence upstream of the splice donor was identified as being required for HIV-2 RNA packaging [8]. To specifically encapsidate the unspliced genomic RNA, HIV-2 has been demonstrated to package its genome in a *cis *rather than a *trans *manner [9]. The structural Gag protein is translated from the full length RNA and encapsidates the RNA from the same pool from which it was translated [8,9].

The *cis *mechanism ensures that only the full length RNA is packaged and provides the specificity. Nonetheless, the requirement for specific sequences implies that an RNA structure is involved in the process. For example, several *in vitro *studies have shown that long-range interactions can regulate RNA encapsidation and dimerisation, both in HIV-1 and HIV-2 [10-13]. Furthermore, a recent study of the HIV-2 5' leader region revealed that an extended stem-loop 1 (SL-1) structure was required for efficient genome encapsidation and viral replication [14].

Dimerisation of retroviral genomes is thought to be linked to the encapsidation process, with elements for both often overlapping [11,15-19]. The HIV-2 leader has been extensively studied and a palindromic sequence within the encapsidation signal has been identified and shown to be important for the regulation of the dimerisation process *in vitro *[11,20]. In addition, several *in vitro *studies proposed that a palindrome located in the loop of SL-1 could act as the dimer initiation site (DIS) [21,22], similarly to what has been shown in HIV-1 [23,24]. To date, the dimeric nature of wild type HIV-2 RNA in cells has yet not been confirmed.

In this study, we analysed a series of SL-1 and Psi mutant viruses, including the 28 nt deletion containing virus mentioned above. Interestingly, viruses deficient for dimerisation *in vivo *were also defective for encapsidation, replication and infectivity. The latter suggests a potential maturation defect and electron microscopy (EM) was performed on virions to determine whether or not this process was affected.

We present here the results of these *in vivo *studies and propose that a previously identified motif, the DIS palindrome, is not required for efficient dimerisation of the HIV-2 RNA in the virus. Our data suggest that genomic RNA dimerisation is mediated by a sequence located within the Psi region and that dimerisation may indeed be closely linked to viral packaging. Importantly, dimerisation defective viruses are deficient in virion maturation and infectivity, potentially offering new targets for inhibiting replication of HIV-2.

## Results

### Mutation of the HIV-2 packaging signal and DIS

**Figure 1:**
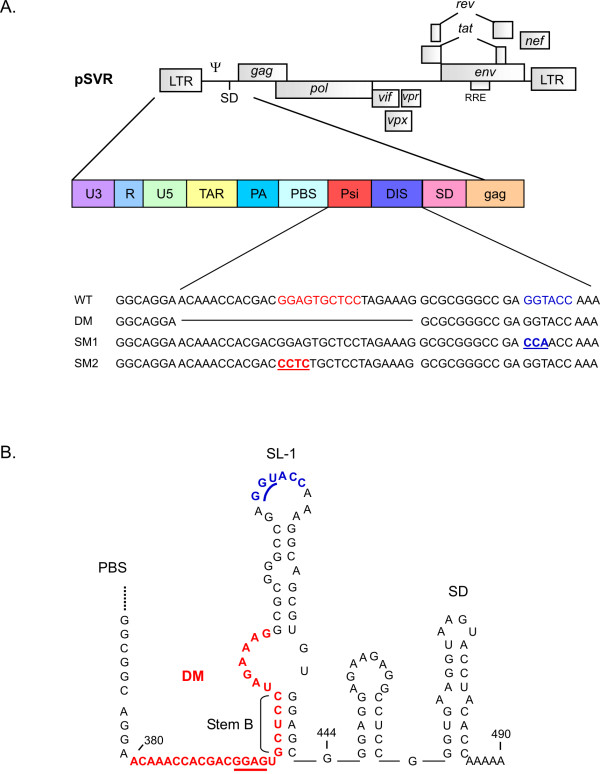
**Genomic and structural context of the mutations introduced in the HIV-2 leader**. (A) Genomic organisation of HIV-2 and location of the mutations introduced. The DM deletion mutant has been described previously [8]. In SM1 the first three bases in the DIS palindrome at position 420 of the HIV-2 RNA genome are substituted. In SM2 the first four bases of the Psi palindrome at position 392 of the HIV-2 RNA genome are substituted. (B) Structure of the SL-1 region predicted by previously published biochemical analyses [20, 22, 55, 56] and *mfold *computer modelling [57, 58. The position of the stem proposed to extend SL-1 is indicated (stem B) [14].

We previously described the DM deletion mutant (Fig. 1A) of HIV-2 which shows a major packaging defect [8]. Recently, the formation of a stem, named stem B and located at the base of SL-1 (Fig. 1B), was shown to be required for efficient genome encapsidation and viral replication [14]. The sequence involved in the formation of stem B is part of a 10 nt palindrome (pal), located within the packaging signal Psi (Ψ), and which has been suggested to play a role in RNA dimerisation *in vitro *[21]. To investigate the requirement for this palindromic sequence in HIV-2 dimerisation *in vivo*, we mutated the first four bases of pal but maintained the bases involved in stem B formation (Fig. 1A, SM2). In HIV-2, initiation of dimerisation has been postulated to occur at the DIS, a palindrome located at the top of SL-1 (Fig. 1B) [21,22]. Interestingly, mutations of the HIV-1 DIS revealed that the sequence was not required for genomic RNA dimerisation *in vivo *or for viral replication in primary cells but was important for replication in T cells [25,26]. Hence, we decided to examine whether the HIV-2 DIS was required for genomic RNA dimerisation and viral replication by mutating the first three bases of the palindrome (Fig. 1A, SM1).

None of the above described mutations had a significant effect on protein production by the virus as judged by western blot analysis (data not shown, [8]), even though the reverse transcriptase (RT) activities of the DM and SM2 mutants were slightly reduced compared to that of the wild type and SM1 mutant (data not shown), suggesting that the virus production is slightly lower for the two Psi mutants.

### Mutations of the HIV-2 packaging signal, but not the DIS, reduce genomic dimerisation *in vivo*

**Figure 2:**
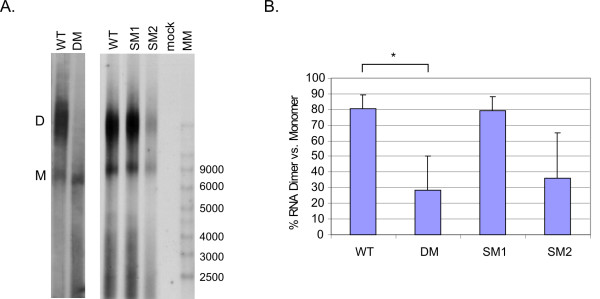
**Mutations in HIV-2 packaging signal, but not in the DIS, render the RNA monomeric *in vivo***. (A) Native northern blot analysis of HIV-2 genomic RNA extracted from pelleted virions 48 h after COS-1 cell transfection. RNA inputs were normalised on RT activity and an equivalent to 2.5 × 10^6 ^cpm was used. WT, wild type; DM, Psi deletion mutant; SM1, DIS mutant; SM2, Psi pal mutant; mock, mock transfection; MM, Millennium RNA Markers (Ambion); M, monomer; D, dimer. (B) Bar chart representing the percentage of dimer present in each virion RNA sample. Data from at least three independent experiments are shown, error bars correspond to the SD. *, Student t test p value < 0.05.

We analysed the effect of the mutations in the packaging signal (DM and SM2) and the DIS (SM1) on genomic RNA dimerisation. Virion RNA extracted from wild type and mutant HIV-2 was analysed by non-denaturing northern blot (Fig. 2A) and the percentage of dimer present in each sample was quantified by densitometry (Fig. 2B). RT activity was measured to load RNA from an equivalent amount of virus particles in each lane. Wild type HIV-2 RNA appeared mostly dimeric within the virion (80%, Fig. 2B), whereas viruses bearing mutations in the Psi region (DM and SM2) showed a significant defect in dimerisation, with around 30 to 35% dimer (Fig. 2B), in addition to a reduction in the apparent amount of RNA encapsidated (Fig. 2A). These results demonstrate that the Psi region contains a signal essential for efficient dimerisation *in vivo *and that at least part of it is mapped to the palindrome pal. Surprisingly, mutation of the DIS palindrome does not have an effect on dimerisation *in vivo *and 80% of the genomic RNA packaged appears dimeric (Fig. 2A and 2B). This result contradicts data obtained in an *in vitro *dimerisation assay, where short RNA transcripts harbouring the SM1 mutation did not dimerise efficiently (data not shown). However, this discrepancy reflects the importance of working in the context of the whole virus, where different long-range interactions and secondary and tertiary structures than those observed *in vitro *[10,12,27] might influence the ability of the genome to form a dimer. Furthermore, a number of factors such as the Gag and nucleocapsid (NC) proteins, which have been shown to promote dimerisation [10,27-30], are only present in the context of the virus.

### Encapsidation efficiencies of the HIV-2 mutants

**Figure 3:**
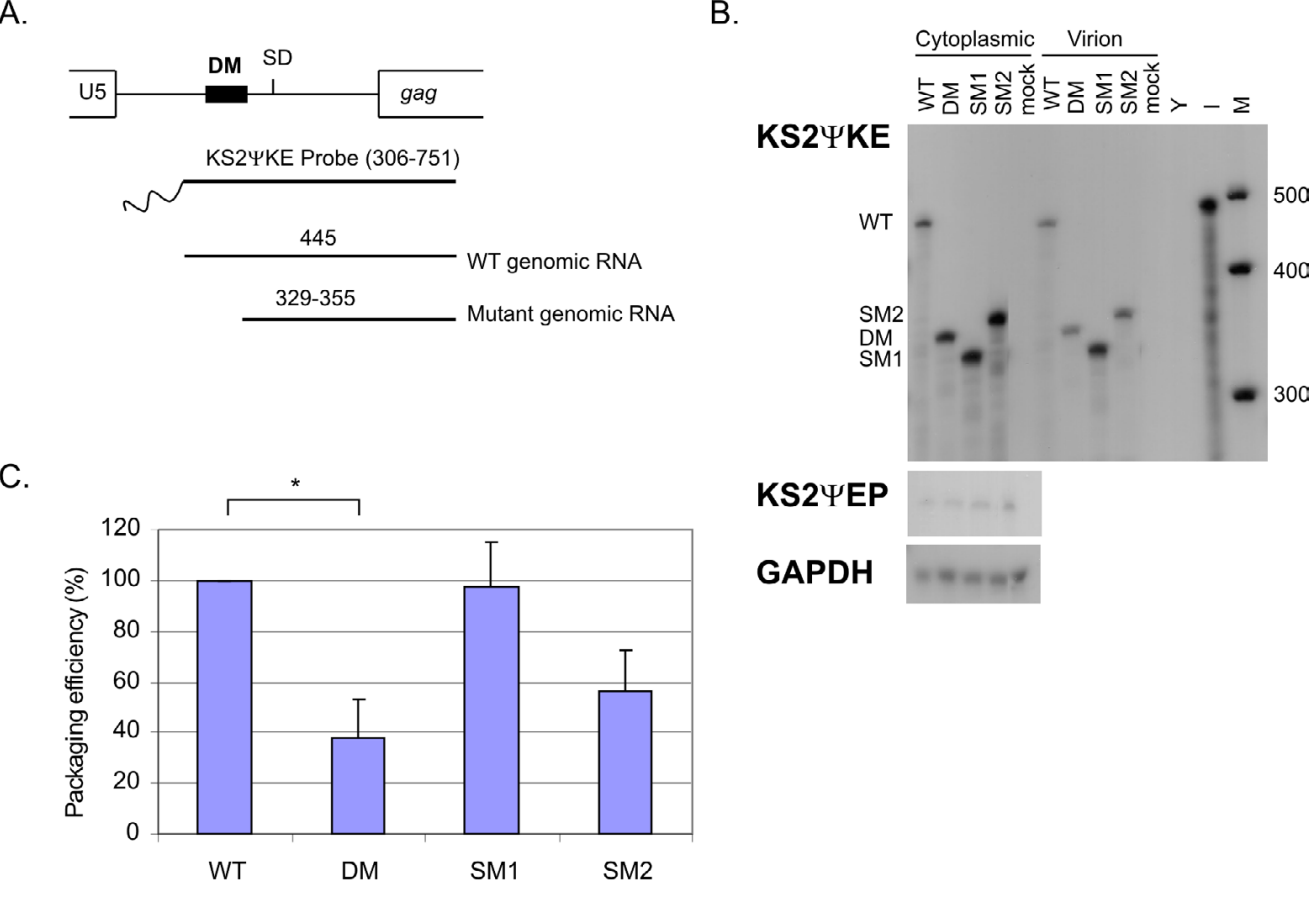
**Encapsidation efficiency of the Psi and DIS HIV-2 mutants**. (A) Size of the protected fragment corresponding to the viral genomic RNA when using the KS2ΨKE riboprobe in an RNAse protection assay (RPA). Protected mutant genomic RNAs vary in size between 329 and 355 nt due to the position of the mutations. (B) Representative RPA where 2 μg of cytoplasmic RNA and an equivalent of 2.5 × 10^6 ^cpm of virion RNA was probed with 1 × 10^5 ^cpm of KS2ΨKE riboprobe. Samples were also probed with 1 × 10^5 ^cpm of KS2ΨEP and GAPDH riboprobes to detect plasmid DNA contamination and control for the loading, respectively. WT, wild type; DM, Psi deletion mutant; SM1, DIS mutant; SM2, pal mutant; mock, mock transfection; Y, yeast RNA + RNase; I, yeast RNA – RNAse (diluted 1:10); M, Century Plus RNA Markers (Ambion). (C) Packaging efficiencies of mutant HIV-2 relative to WT virus. Data from 3 independent experiments are shown, error bars correspond to the SD. *, Student t test p value < 0.005.

Deletion of the DM sequence (Fig. 1A) was previously reported to cause a severe packaging defect in HIV-2 [8]. Northern blot analysis of the SM2 mutant revealed a possible defect in encapsidation (Fig. 2A), even though only four bases of the Psi region are substituted in this mutant. To investigate this further, we assessed the level of HIV-2 genomic RNA in the cytoplasm of transfected cells and pelleted virions using RNase Protection Assay (RPA, Fig. 3). Wild type and mutant genomic RNAs were detected by probing with KS2ΨKE (Fig. 3B) and the size of the protected fragments are shown in figure 3A. A specific riboprobe (KS2ΨEP) was used to measure plasmid DNA contamination (Fig. 3B). Finally, equal loading of cytoplasmic RNA was confirmed by probing for GAPDH mRNA (Fig. 3B). Packaging efficiencies, taken as the ratio of virion to cytoplasmic RNA of a mutant relative to the wild type, are reported in figure 3C. As observed in figure 2A, the SM2 mutant displayed some reduction in packaging (approx. 40% on average). Although this figure is lower than for the DM deletion mutant, which showed a 70% decrease in RNA encapsidation, statistical analysis revealed that the difference in the packaging efficiencies of these two mutants was not significant. By contrast, the SM1 mutation of the DIS did not affect HIV-2 RNA encapsidation (Fig. 3C).

### Viruses with mutations in the packaging signal and impaired dimerisation fail to replicate in T cells

**Figure 4:**
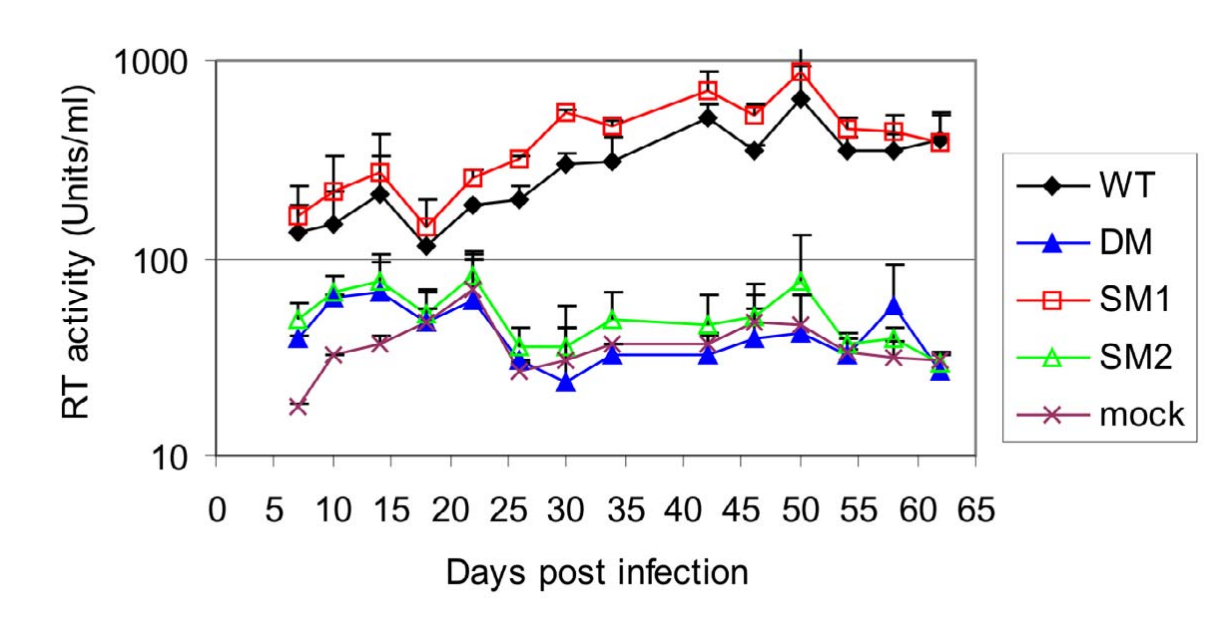
**Mutations in the HIV-2 packaging signal affect replication of HIV-2 in T-cells**. 1 × 10^6 ^Jurkat T-cells were infected with an amount of virus equivalent to 1 × 10^7 ^cpm as measured by RT activity assay. Replication was assessed by measure of the RT activity every 4 days. Cells were passaged 1/2 every 8 days but no new cells or viruses were added at any point during the course of the infection. Data from two independent experiments are shown. Error bars represent the SD.

Since the SM2 mutant exhibited a comparable reduction in the level of dimer as the DM mutant, but retained a greater portion of the HIV-2 packaging determinant and possibly encapsidated its genome more efficiently than the DM mutant, it was of interest to compare the replication kinetics of these two mutants (Fig. 4). Despite having no defect in RNA encapsidation or dimerisation, the SM1 mutant was included to verify that mutation of the DIS does not impair viral replication over a longer period of time. As shown on figure 4, viral spread in the DM and SM2 mutants was markedly reduced and the mutant viruses were not able to revert to a replication competent phenotype despite prolonged culture. The similar behaviour of the DM and the SM2 mutants suggest that a failure to encapsidate a dimeric genome rather than just a reduction in encapsidation might be responsible for the replication defect observed. Indeed, viral RNA dimerisation has been associated with viral infectivity in other retroviruses [16,19,31,32]. The SM1 mutant virus replicated as efficiently as wild type virus, confirming that an intact DIS palindrome is dispensable for the establishment of a productive infection.

### Dimerisation deficient viruses display a reduced infectivity disproportionate to the packaging defect

**Figure 5:**
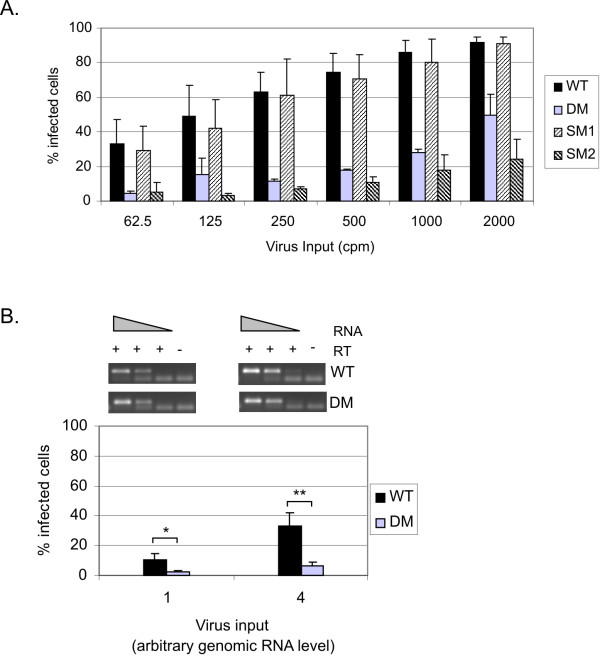
**Virus infectivity is decreased by mutations in the Psi region**. (A) Viral infectivity was determined using a reporter cell line expressing the *gfp *gene under the control of the HIV-2 LTR (Ghost CCR5/CXCR4). Equivalent amounts of VSV-G pseudotyped wild type (WT) and mutant HIV-2, normalised on RT activity, were used to infect 5 × 10^3 ^Ghost CCR5/CXCR4 cells and expression of GFP was measured at 72 h post-infection by FACS. Data from at least three independent experiments are plotted. Error bars represent the SD. (B) Virus inputs were adjusted according to the DM virus packaging defect as to infect the Ghost cells with an equivalent level of genomic RNA for WT and DM virus. Top panel: HIV-2 genomic RNA was extracted from two set amounts of virions used to infect the Ghost cells and viral RNA levels were assessed by semi-quantitative RT-PCR using neat, 1:10 and 1:100 dilution of the RNA sample. A GAPDH carrier RNA was added during the extraction and amplified in parallel by RT-PCR to control for RNA loss during the RNA extraction (not shown). A control without RT was performed on 10 μl of neat RNA (-). Bottom panel: The percentage of GFP positive cells was measured by FACS at 72 h post-infection. Arbitrary levels of RNA of 1 and 4 are equivalent to 62.5 cpm and 250 cpm of WT virus, respectively, as measured by RT activity assay. Data from four independent experiments are shown on the chart. Error bars represent the SD. *, Student t test p value < 0.05; **, p value < 0.01.

Although we now had evidence that mutation of the DM region, including pal, altered both genomic RNA encapsidation and dimerisation, it was important to know whether this alone was responsible for the very poor replication of the DM and SM2 viruses in T cells. Hence, we examined whether the mutant viruses were impaired in infectivity using the Ghost CCR5/CXCR4 reporter cell line which contains a stably transfected reporter cassette, consisting of an HIV-2 LTR driving expression of a green fluorescent protein (*gfp*) gene. Successful infection of these cells by HIV-2 leads to activation of the cassette by the newly synthesised Tat protein and can be detected by measuring GFP production in the infected cells. We compared the infectivity of VSV-G pseudotyped wild type and mutant virions. Both the DM and the SM2 mutants showed a strikingly lower level of infectivity when equivalent amounts of virus, as measured by RT activity, were used to infect the Ghost cells (Fig. 5A). As observed in T cells, the SM1 mutation had no effect on the ability of the virus to infect permissive target cells. The envelope-deleted viruses or VSV-G envelope had no effect on GFP expression when the corresponding plasmids were transfected individually into COS-1 cells (data not shown). The VSV-G envelope was used to pseudotype envelope-deleted HIV-2. This may influence the ability of the virus to enter and hence infect the target cells because of the postulated difference in entry mechanism using this envelope. However, a preliminary experiment was carried out using full-length wild type and DM mutant HIV-2. Hela cells stably transfected with a reporter cassette, consisting of an HIV-1 LTR driving expression of a beta-galactosidase gene, were used. Similar results as those observed in the Ghost CCR5/CXCR4 cells were obtained (data not shown), even though the high level of background staining rendered the determination of an exact infectious titre difficult.

The reduced infectivity observed could be due to the defective packaging of the DM and SM2 viruses and thus the delivery of fewer genomes to the Ghost cells. Therefore, we were concerned to ensure that the absolute quantity of genomic RNA delivered to the Ghost cells was similar for wild type and mutant viruses. Since we have shown that the DM deletion virus displayed a three fold reduction in RNA encapsidation (Fig. 3C), we augmented the DM virus input by three fold so that a comparable number of genomes would be delivered to the Ghost cells (Fig. 5B). We verified the level of HIV-2 genomic RNA in two viral inputs by semi-quantitative RT-PCR performed on serial dilutions of the virus inoculum (Fig. 5B, top panel). To ensure that the RNA extraction process did not result in a loss of RNA, leading to differences in genomic RNA levels, a GAPDH carrier RNA was added prior to the extraction and analysed in parallel by RT-PCR as an internal control (data not shown). Interestingly, targeting equivalent numbers of HIV-2 genomes to the Ghost cells did not lead to equivalent infectivity (Fig. 5B). With both inputs tested, infectivity of the DM virus was three to five fold lower than that of the wild type virus. These results show that the infectivity defect seen in figure 5A was not simply a consequence of reduced numbers of virus genomes entering the Ghost cells.

### Encapsidation of a dimeric genome is important for HIV-2 particle maturation

**Figure 6:**
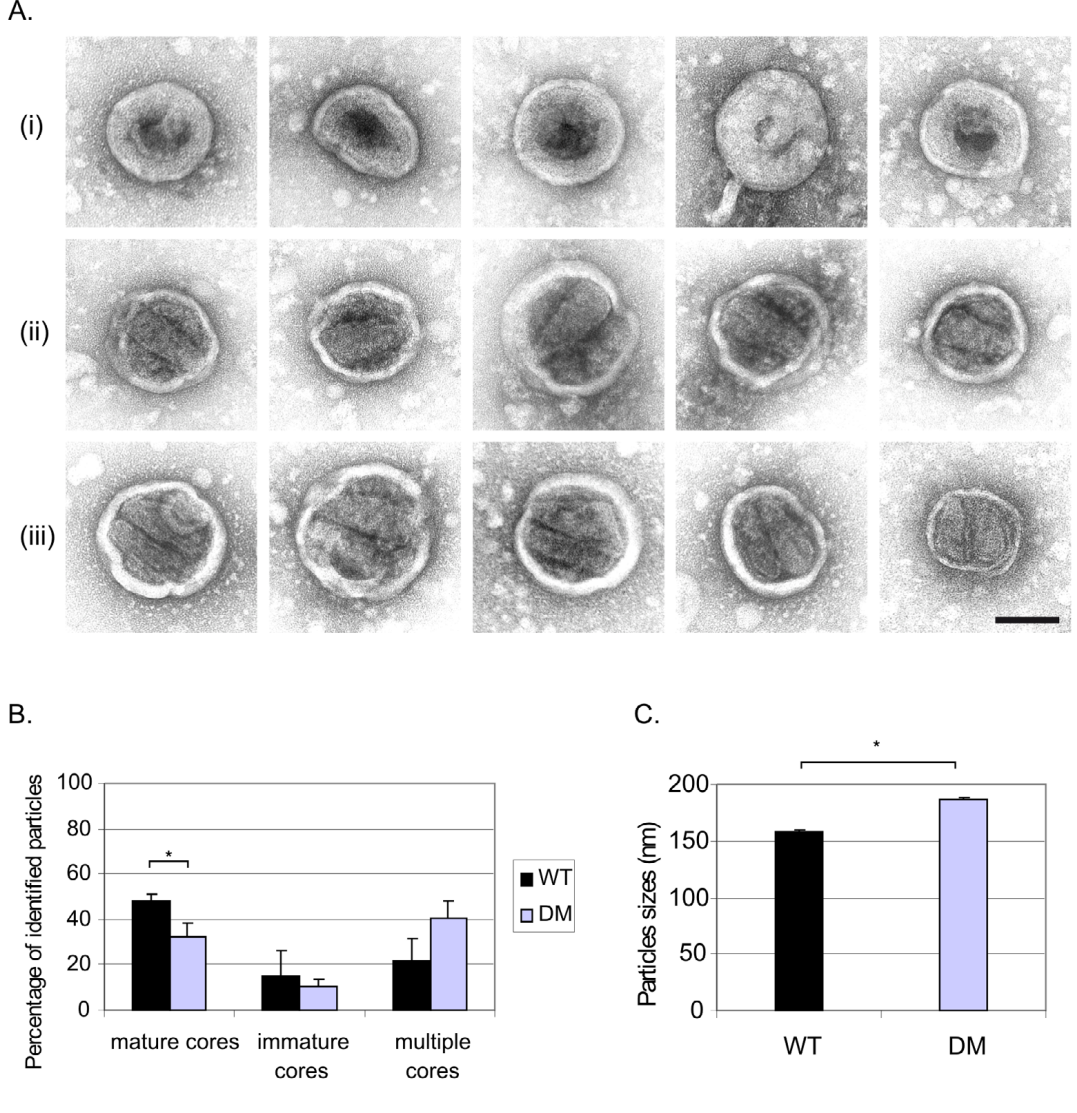
**Efficient dimerisation and encapsidation are associated with correct assembly and particle maturation in HIV-2**. COS-1 cells were transfected with envelope-deleted provirus and virions were harvested 48 h later. Purified HIV-2 particles were analysed by negative staining and electron microscopy. (A) Five examples of what were assessed as immature particles (i), mature particles (ii) and particles containing multiple cores (iii) are shown. Scale bar: 100 nm. (B) The proportion of mature, immature and multiple core particles was calculated for wild type (WT) and DM mutant HIV-2 after examination of 193 WT and 199 DM particles by two individuals independently. Error bars correspond to the SD. *, Student t test p value < 0.05. (C) The particles were measured and the average sizes of WT and DM particles were reported on the graph. Error bars correspond to the SD; n=215 for WT and 189 for DM; *, Student t test p value < 0.05.

To investigate whether there was any contribution of RNA encapsidation and genome dimerisation to viral assembly in HIV-2, we purified virions produced by cells transfected with either wild type or DM deletion provirus, both envelope-deleted, and analysed them using electron microscopy and negative staining. Electron micrographs were examined by two independent observers and approximately two hundred particles were identified for each virus. Representative particles containing immature, mature and multiple cores are shown in figure 6A (i), (ii) and (iii), respectively. We observed that cells transfected with the DM proviral DNA produced significantly fewer mature particles as compared to those transfected with wild type provirus (Fig. 6B). Surprisingly, the loss of mature particles for the DM virus was not accompanied by an increase in the proportion of immature virions (Fig. 6B). However, there was a larger number of duplex cores in the DM deletion virions, although this difference does not appear to be statistically significant (Fig. 6B). Furthermore, the existence of multiple cores in mature virions has been documented previously for the related virus HIV-1 [33]. These results show that a mutation in the HIV-2 packaging signal that also affects genome dimerisation leads to a reduction in the number of mature particles and an abnormal proportion of particles containing more than one core, possibly explaining the loss of infectivity associated with this mutation. Finally, it was also notable that the average diameter of the mutant virions was larger than that of the wild type virions (Fig. 6C). This did not appear to solely result from an increased number of particles containing multiple cores, unlike in HIV-1, where particles containing two cores were significantly larger than those containing a single core [33]. Taken together, these results suggest that genomic RNA encapsidation, genome dimerisation and virion particle morphology are extremely closely linked in HIV-2.

## Discussion

We have explored the SL-1 region of the HIV-2 5' leader and shown that a signal essential for *in vivo *dimerisation in the viral particle is disrupted when the packaging signal is mutated. Substitution of four nucleotides in the Psi palindrome is sufficient to partially impair dimerisation and encapsidation of the virus, suggesting that the two processes may be linked. This result confirms a previous report showing that the Psi palindrome is involved in RNA dimerisation in HIV-2 [11,20].

Deletion of the whole Psi region significantly reduces the level of dimeric genome in the virions, in addition to causing a strong encapsidation defect.

While both Psi mutants exhibited a similar decrease in the percentage of dimer present (30% and 35% for DM and SM2, respectively), a large variation was observed. This reflects the difficulty of quantifying the dimeric RNA bands using densitometry. Indeed, the RNA dimer does not migrate as a distinctive neat band but rather as a variably spread band. In addition, it is not always possible to clearly distinguish the end of the dimer band from the beginning of the monomer band and the background in between. This is not a problem when analysing a virus that dimerises well, as the very strong intensity of the dimer band renders it easier to delimit and quantify (e.g. WT and SM1, Fig. 2A). Hence, even though the variation may appear quite large for the DM and the SM2 mutants, both viruses exhibited a significant reduction in the level of dimeric RNA packaged.

Similarly, although the defect in genomic RNA encapsidation observed for the DM mutant appears more pronounced than that observed for the SM2 mutant, both mutants encapsidated significantly less RNA than the wild type virus. Since only four bases were mutated in the SM2 virus as compared to a deletion of 28 nt in the DM virus, it might explain the difference in the packaging efficiencies of DM and SM2. Furthermore, the stability of the genomic RNA dimer of the DM and SM2 mutant may not be the same and could influence the ability of these viruses to encapsidate their genomes. Further work in our laboratory will aim to determine if the different mutations introduced in the 5' UTR of HIV-2 affect the stability of the RNA dimer. This will help establish whether the same mechanism is involved in the reduction of viral infectivity observed with the DM and SM2 mutants.

The RNA dimer was shown to undergo protease-dependent maturation in Murine leukaemia virus (MuLV) and HIV-1 [30,34], although it was later demonstrated that Gag cleavage alone was not sufficient to promote dimer maturation [35]. The proteolytic processing of the p2/CA cleavage site and the spacer peptide p1 have both been implicated in the stability and maturation of the HIV-1 RNA dimer [36,37]. To date, it remains unclear as to whether the reduction in the level of dimer detected in the DM and SM2 mutant virions results from a defect in the dimer formation or a decrease of the dimer stability.

A recent study of HIV-1 dimerisation proposed that genomic RNA is packaged as a monomer and that dimerisation subsequently occurs at the DIS in a three-step protease-dependent mechanism [38], although several other studies in HIV-1 and MuLV suggested that dimerisation might have already occurred within the infected cell [15,17,39,40]. Therefore, it would of interest to analyse whether the formation of dimeric HIV-2 genomic RNA occurs prior to encapsidation or involves a protease-dependent step.

The DM mutant shows a defect in infectivity that is independent of the reduction in encapsidation, as targeting a similar amount of genome to the target cells does not restore infectivity. Furthermore, both viruses mutated in the Psi region were unable to establish a productive infection in T cells, suggesting that encapsidation of a dimeric genome might be required for viral infectivity in HIV-2. This involvement of SL-1 in RNA encapsidation and dimerisation has been reported for simian immunodeficiency virus macaque (SIV_mac_), where mutations in this region also resulted in a reduction in viral infectivity [19].

Interestingly, introduction of a mutation in the DIS palindrome so that it was no longer self-complementary did not yield any decrease in the level of dimeric genome *in vivo*. This result contradicts previous *in vitro *studies where an SL-1-mediated dimerisation [21,22] was proposed to be regulated by several long-range interactions [10,11,27]. However, in the context of the virus, different interactions and conformational changes might occur and *trans*-acting factors such as the Gag protein have been shown to promote dimerisation in HIV-1 and MuLV [28,29]. Furthermore, in HIV-1, the DIS palindrome was also reported to be dispensable for dimerisation *in vivo *[25] and replication in primary cells [26]. However, an intact HIV-1 DIS was required for efficient RNA packaging and viral replication in T cells [16,25,32], which we did not observe in HIV-2. This suggests that the palindrome termed the DIS in HIV-2 is not an important Dimer Initiation Site *in vivo*, even though it might be involved at some stage of the dimer formation.

In the mutants of the Psi region studied here, there was a correlation between an encapsidation and a dimerisation defect, indicating that the two processes may be closely linked, as previously proposed for HIV-1, HIV-2 and MuLV [11,16,25,32,40]. This suggests either that one is dependent on the other or that the same or closely adjacent sequences are responsible for both processes.

The dimeric RNA genome has been shown to have a number of different roles in several stages of the retroviral life cycle, such as recombination during reverse transcription [39,41] and proviral DNA synthesis [16,42,43]. In addition, RNA was proposed to have a structural role in MuLV [44]. We have now shown that, in HIV-2, the absence of a dimeric genome is associated with a severe replication defect which is disproportionately large compared to the diminution in RNA encapsidation. Packaging of monomeric genomes occurs but is less efficient than that of dimeric ones. However, delivery of viruses containing monomeric genomes to target cells is characterised by a reduction in infectivity which cannot be restored simply by increasing the number of genomes delivered. Thus, it seems that a monomeric genome impairs virus viability.

Previous studies have shown that RNA capture influences viral assembly by both affecting the efficiency and contributing to normal capsid morphology [1,19,44,45]. In SIV, mutations in the SL-1 region that resulted in a reduction of encapsidation and dimerisation also led to aberrant particle morphology [19]. EM of our viruses containing monomeric genomes shows that there is a significant reduction in particle maturation compared to when the dimeric genome is present and that larger cores can be detected. However, the proportion of immature particles in the mutant virus sample is similar to that of the wild type virus preparation, indicating that the mutant viruses undergo at least some degree of maturation. Furthermore, protein processing of the DM mutant appeared normal when analysed at the same time point of 48 hrs post-transfection that we used in this study [8]. Nonetheless, a delay in protein processing cannot be excluded as the analysis was only performed at a single time point. In HIV-1, mutations of the DIS were reported to cause a delay in the processing of the p2 peptide [46], which has been demonstrated to be involved in the sequential proteolytic processing of the Gag protein [47]. In addition, RNA was proposed to be required for the cleavage of the HIV-1 NCp15 precursor [48], suggesting that binding of the nucleocapsid protein to the RNA might play a role in virion maturation. With this in mind, it would be interesting to analyse the protein processing of the mutant virus at several time points and determine whether the processing of the Gag and Gag-Pol polyproteins is affected.

Intriguingly, there is an increased number of particles containing duplex cores in the DM deletion virions. This phenomenon has previously been observed in HIV-1, where approximately 30% of the particles contained two cores [33]. Since we have shown here that the DM virions contain predominantly monomeric RNA, it is tempting to speculate that these cores each contain a single monomeric genome, although proving this rigorously will be challenging.

Our work is consistent with dimeric RNA being involved in efficient packaging in HIV-2. In MuLV, recent structural studies have implied that dimerisation of the genomic RNA leads to exposure of a high affinity Gag binding site *in vitro *[15], suggesting that dimeric RNA is important for the Gag:RNA interaction. Therefore, it would be of great interest to analyse whether our mutations in the SL-1/Psi region affect the binding affinity of the Gag protein to the RNA.

It remains to be seen whether the encapsidation process in other Lentiviruses, and particularly HIV-1, is also dependent on formation of an RNA dimer prior to Gag capture. Molecules which interfere with this process might be exploited as antiviral agents and would be expected to have a high degree of selectivity for the virus, since RNA export is virus specific in animal cells and export of a dimeric species is, as far as we know, unique to retroviruses. Indeed, there is recent evidence that oligonucleotides targeting the dimer linkage of HIV-1 are effective antiviral agents *in vitro *[49].

## Conclusion

In this study, we have shown that in HIV-2, the *cis*-acting packaging and dimerisation elements are closely linked and may be part of the same structural region of the 5' UTR. Surprisingly, the DIS does not mediate HIV-2 genomic RNA dimerisation *in vivo *and does not seem to play a significant role in viral replication or infectivity. Packaging a monomeric genome appears to adversely affect the production of infectious viral particles and the virions that are produced have an abnormal morphology. It therefore seems likely that the dimeric genome plays an important role in the virion, both from its coding potential but also as a structural element ensuring optimal virus assembly.

## Methods

### Plasmid construction

pSVR is an infectious proviral clone of HIV-2 ROD containing a simian virus 40 origin of replication [7]. Restriction sites and nucleotide numbering, where given, are relative to the first nucleotide of the viral RNA. Proviral construct pSVRDM containing a 28 nt deletion in the 5' leader has been previously described [8]. Mutations in the 5' leader were introduced by site-directed mutagenesis [50] into a subclone of HIV-2, pGRAXS [8], using primers 5' GGCGCGGGCCGACCAACCAAAGGCAGCGTGTGG 3' and its complement and 5' GGAACAAACCACGACCCTCTGCTCCTAGAAAGGCG 3' and its complement for SM1 and SM2, respectively. Sequences from the resulting subclones were introduced into the provirus by exchanging an *Aat*II-*Xh*oI fragment, generating proviral constructs pSVRSM1 and pSVRSM2. Proviral constructs pSVRΔNB and pSVRΔNBDM which contain a 550 nt deletion in the *env *gene (positions 6369 to 6927) have been previously described [8]. pSVRΔNBSM1 and pSVRΔNBSM2 were generated by replacing the *Aat*II-*Xh*oI region of pSVRΔNB with the same region of pSVRSM1 and pSVRSM2 respectively. pCMV-VSVG contains the vesicular stomatitis virus (VSV) G glycoprotein gene in the context of pCDNA3 (Invitrogen). All plasmids based on HIV-2 proviral sequences were grown in TOPF'10 (Invitrogen) *Escherichia coli *at 30°C to avoid recombination. All other plasmids were grown in DH5α *E. coli *under standard conditions.

Plasmids used as template for production of riboprobes were constructed as follows. Plasmids KS2ΨKE and KS2ΨEP have been previously described [8,51]. They contain HIV-2 sequences from positions 306 to 751 and (-107) to 306 of pSVR, respectively, cloned into the polylinker of the pBluescript KSII(+) transcription vector (Stratagene). A glyceraldehyde-3-phosphate dehydrogenase (GAPDH) riboprobe template was created by reverse transcriptase (RT)-PCR amplification of the sequence from position 61 to 346 of human GAPDH using 5' GGTGAAGGTCGGAGTCAACG 3' and 5' AATTAACCCTCACTAAAGGACTCCACGACGTACTC 3' primers. Total cytoplasmic RNA extracted from Jurkat T cells served as template for the reaction. *In vitro *transcription of the linearised plasmids and PCR fragment using T3 RNA polymerase yielded antisense riboprobes for use in the northern blot and RNase protection assay (RPA).

### Cell culturing and transfection

COS-1 simian epithelioid cells, obtained from the European Collection of cell culture (ECACC), were maintained in Dulbecco's modified Eagle's medium (DMEM, Gibco BRL) supplemented with 10% fetal calf serum and 1× penicillin/streptomycin (Invitrogen). Cells were transfected in 10 cm-diameter dishes by the DEAE-dextran method [52] with a total of 5 μg of DNA. Cells and supernatants were harvested 48 h later, and virus production was assessed with a RT assay [53]. CD4^+ ^human osteosarcoma (HOS) cells stably transfected with CCR5, CXCR4 and the green fluorescent protein (*gfp*) reporter gene under the control of HIV-2 LTR (designated Ghost CCR5/CXCR4), provided by the Centralised Facility for AIDS Reagents, NIBSC, UK, were maintained in DMEM supplemented with 10% fetal calf serum, 1× penicillin/streptomycin (Invitrogen), 100 μg/ml hygromycin (Invitrogen), 500 μg/ml geneticin (Gibco BRL) and 1 μg/ml puromycin (Sigma) as previously described [54]. Jurkat T cells, provided by the Centralised Facility for AIDS Reagents, NIBSC, UK, were maintained in RPMI 1640 medium (Roswell Park Memorial Institute, Gibco BRL) supplemented with 10% fetal calf serum and 1× penicillin/streptomycin (Invitrogen).

### T-cell replication assay

Replication assays were performed as described previously [8] with the following modifications. Pelleted virus from two individual transfections were resuspended in 500 μl RPMI 1640 each and pooled. Subsequently, an amount of virus equivalent to 1 × 10^7 ^cpm of RT activity was added to 1 × 10^6 ^Jurkat cells in the presence of 10 μg/ml DEAE-dextran in one well of a 6-well culture plate. After 24 h at 37°C, cells were transferred into a 25 cm^2 ^flask containing 10 ml of media and virus replication was followed by measuring RT activity every 4 days. Cells were split 1/2 every 8 days.

### Transduction of HOS-CD4^+ ^LTR-GFP cells

Supernatants of COS-1 cells transfected with 5 μg envelope-deleted provirus and 5 μg VSV-G expressor plasmid, 10 μg envelope-deleted provirus alone or 10 μg VSV-G expressor alone as well as mock transfected cells were harvested as previously described [8] except that pelleted virus from two individual transfections were resuspended in 500 μl DMEM each and pooled. The RT activity of the resulting virus preparation was determined as described above and the amount of virus added to a 6-well plate of cells was normalised this way. Ghost CCR5/CXCR4 cells were seeded at 2 × 10^5 ^cells per well in selection medium 24 h prior to infection. Cells were exposed to increasing amount of WT and mutant viruses in the presence of 8 μg/ml polybrene (Sigma) for 16 h, after which the virus-containing medium was replaced by fresh selection medium. At 72 h post-infection, cells were washed twice with cold PBS, trypsinised and fixed in 2% paraformaldehyde. GFP expression was measured by FACS analysis.

### RT-PCR

Viral RNA from normalised amounts of virions was isolated using the QiAamp viral RNA kit (Qiagen). 1 μg of *in vitro *synthesised GAPDH RNA (pos. 61 to 346) was added to each sample prior to extraction. The extracted RNA was treated with 5 U TURBO DNase (Ambion) for 1 h at 37°C, followed by heat inactivation for 10 min at 65°C. 10 μl of neat RNA, 1:10 or 1:100 dilution were used for RT-PCR using One step RT-PCR kit (Abgene) with HIV-2 Psi F 5' TAATACGACTCACTATAGGCTGAGTGAAGGC 3' and Psi R 5' AGGTACTTACCTTCACCC 3'. A control without RT was performed with 10 μl of neat RNA. Samples were visualised on a 2% agarose gel.

### RNA isolation

Cytoplasmic and virion RNAs were harvested with the RNAeasy mini kit (Qiagen) and the QIAamp viral RNA kit (Qiagen), respectively. The isolated RNA was treated with 5 U of TURBO DNase (Ambion) for 30 min at 37°C and extracted once with acid-buffered phenol-chloroform and once with chloroform. RNA was precipitated with ammonium acetate and ethanol and stored at -80°C.

### Northern blot analysis

KS2ΨKE biotinylated riboprobe was synthesised by *in vitro *transcription of linearised plasmid using T3 RNA polymerase (Promega), and purified with a G-50 column (Roche) prior to use in northern blot assays. Reagents for the northern blots were obtained from a commercially available kit (Ambion). Virion RNA input was normalised on RT activity, with an equivalent of 2.5 × 10^6 ^cpm being the standard amount used per reaction. RNAs were subjected to non-denaturing electrophoresis on 0.8% LE-agarose (Ambion) gel. For size determination, biotinylated RNA Millennium markers (Ambion) were run in parallel. RNAs were transferred onto positively charged nylon membranes (Ambion) prior to incubation with 0.4 nM riboprobe for 16 h at 68°C. Detection was performed using the BrightStar Biodetect kit (Ambion). RNA was quantified by densitometry using the ImageJ software.

### RNase protection assays

[α-^32^P]-labelled KS2ΨKE riboprobe was synthesized by *in vitro *transcription of linearised plasmid using T3 RNA polymerase (Promega) and gel purified prior to use in RPA, as described previously [8]. RNA inputs were normalised on concentration, for cytoplasmic RNA, and RT activity, for virion RNA. Typically, 2 μg of cytoplasmic RNA and an equivalent of 2.5 × 10^6 ^cpm of viral RNA were used. For each experiment, a separate RPA was performed using the same RNA inputs but probing for viral plasmid DNA using a probe generated from plasmid KS2ΨEP. In addition, a probe for human GAPDH RNA was included in the reaction to control for variations in cytoplasmic RNA input. Any DNA contamination or variations in the GAPDH signal were accounted for when calculating encapsidation efficiencies, taken as the ratio of virion to cytoplasmic RNA of a mutant relative to the wild type.

### Electron microscopy

Supernatants of COS-1 cells transfected with envelope-deleted WT and DM deletion mutant proviruses were harvested as previously described [8]. Pelleted virions were resuspended in 10 μl of 100 mM NaCl, 50 mM MOPS buffer at 4°C for 24 h prior to negative staining. 0.8 μl of virion preparation was applied to a carbon coated grid and stained with a few drops of 1% uranyl acetate. Micrographs were recorded on a Philips EM208S at a nominal magnification of × 20,000.

## Competing interests

The author(s) declare that they have no competing interests.

## Authors' contributions

AL, JSG and AML designed the study. AL carried out the experiments. RAC performed the EM experiment. AL, JSG and AML drafted the manuscript. All authors read and approved the final manuscript.
